# Case Report: Hypothyroidism Misdiagnosed as Fulminant Myocarditis in a Child

**DOI:** 10.3389/fcvm.2021.698089

**Published:** 2021-06-11

**Authors:** Nanjun Zhang, Shuran Shao, Yu Yan, Yimin Hua, Kaiyu Zhou, Chuan Wang

**Affiliations:** ^1^Department of Pediatric Cardiology, West China Second University Hospital, Sichuan University, Chengdu, China; ^2^West China Medical School of Sichuan University, Chengdu, China; ^3^The Cardiac Development and Early Intervention Unit, West China Institute of Women and Children's Health, West China Second University Hospital, Sichuan University, Chengdu, China; ^4^Key Laboratory of Birth Defects and Related Diseases of Women and Children (Sichuan University), Ministry of Education, Chengdu, China; ^5^Key Laboratory of Development and Diseases of Women and Children of Sichuan Province, West China Second University Hospital, Sichuan University, Chengdu, China

**Keywords:** children, hypothyroidism, atrioventricular block, fulminant myocarditis, cardiogenic shock

## Abstract

**Background:** Hypothyroidism can lead to bradycardia, reduced cardiac output, cardiac enlargement, and abnormal electrocardiogram. However, hemodynamic instability and malignant arrhythmias due to hypothyroidism is rarely reported in children.

**Patient Findings:** We report the case of a child with third-degree atrioventricular block, cardiogenic shock, and Adams Stokes Syndrome, who was initially misdiagnosed with fulminant myocarditis and was later found to have hypothyroidism during treatment.

**Summary:** The child's condition did not improve after the administration of gamma globulin, methylprednisolone, and isoproterenol. Even after the placement of temporary pacemakers, the therapeutic effect was still not ideal. Upon reviewing the medical history, the child's condition improved rapidly after levothyroxine supplementation.

**Conclusions:** Hypothyroidism is a common disease, but secondary severe cardiovascular lesions are particularly rare in children. Therefore, the delay in diagnosis can lead to serious cardiovascular manifestations. When pediatric patients develop severe AVB and bradycardia, hypothyroidism should be considered as a possible cause.

## Background

Thyroid hormone (TH) is the basic hormone that maintains the functional activity of the body, regulates metabolism and growth in many ways, and affects the function of almost all organs and systems to varying degrees. In cardiovascular terms, hypothyroidism often leads to bradycardia; in severe cases, it could lead to dull heart sound, decreased cardiac output, cardiac enlargement, and abnormal electrocardiogram (ECG). In recent years, some authors have reported cases of advanced atrioventricular block (AVB) caused by hypothyroidism in adults, which improved after the administration of TH (1–5). However, hemodynamic instability and malignant arrhythmias caused by hypothyroidism, which develop into cardiogenic shock and Adams Stokes Syndrome, are rarely reported in children.

Fulminant myocarditis (FM) is an uncommon syndrome characterized by sudden and severe diffuse cardiac inflammation, often leading to death from cardiogenic shock, ventricular arrhythmias, or multiorgan system failure (6). The diagnosis of FM always needs to exclude other diseases such as congenital coronary artery anomalies, cardiomyopathy, rheumatic heart disease, and congenital AVB, since they may share manifestations. However, congenital hypothyroidism is often overlooked as a differential diagnosis for FM in the clinic; the presence of third-degree AVB, cardiogenic shock, and Adams Stokes Syndrome have rarely been observed in patients with congenital hypothyroidism.

We report for the first time in the Chinese population a child with third-degree AVB, cardiogenic shock, and Adams Stokes Syndrome, who was initially misdiagnosed with FM and was later found to have hypothyroidism during treatment. This case report will improve the understanding of the serious cardiovascular complications caused by hypothyroidism. In addition, we suggest that hypothyroidism should be considered as a differential diagnosis of FM.

## Main Text

### Case Report

A 6-year-old boy was urgently admitted to local hospital for frequent convulsions with loss of consciousness, and he had no symptoms prior to the onset (fever, shortness of breath, cough, headache, etc.). The initial ECG showed a third-degree AVB. Transthoracic echocardiography showed an enlarged left ventricle, mild mitral, tricuspid regurgitation, and normal left ventricular systolic function with an ejection fraction (EF) of 58% and a fractional shortening (FS) of 33%. He was primarily diagnosed with FM and was treated promptly with intravenous methylprednisolone (10 mg/Kg per day) and isoproterenol (0.05 ug/Kg/min). Unfortunately, the patient developed persistent abdominal pain, accompanied by vomiting during treatment, followed by sudden respiratory and cardiac arrest. After successful cardiopulmonary resuscitation, the child's heart rate fluctuated between 40 and 60 beats/min, and he was immediately transferred to our hospital.

**Figure 1:**
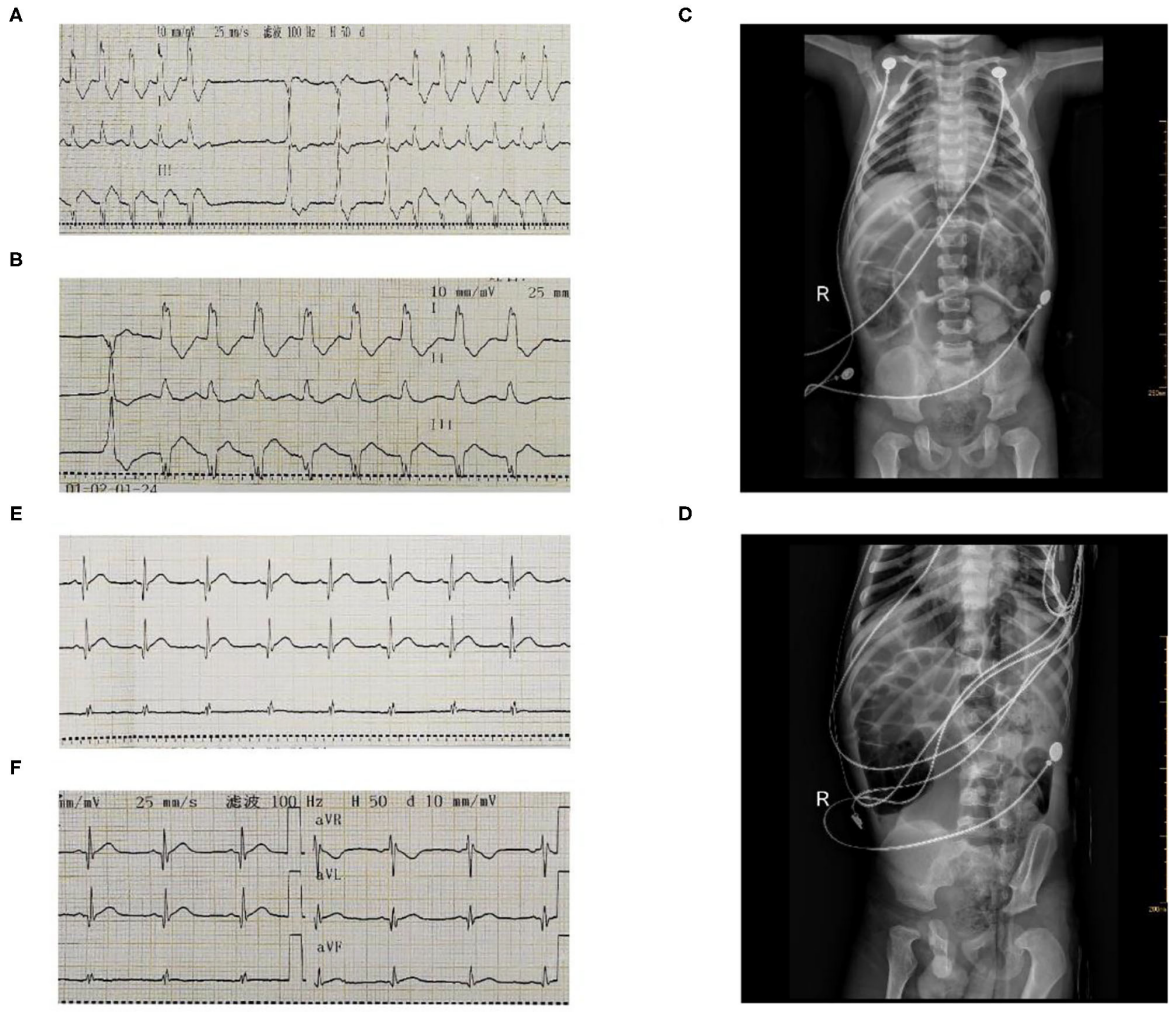
The ECG and chest radiograph of the patient before thyroxine supplementation **(A–D)**. Follow-up with cardiologist and endocrinologist for repeat ECG in 4 weeks **(E,F)**.

The patient was conscious upon arrival to the emergency department in our hospital. Physical examination showed a heart rate of 47 beats/min, respiratory of 23 beats/min, blood pressure of 82/37 mmHg, and temperature of 36.5°C, SpO_2_ of 95%. His face was pale, a dull heart sound and third-degree systolic bruits in the fourth intercostal space could be heard, and abdomen was distent. The ECG showed a third-degree AVB with a minimum heart rate of 75 beats/min (on isoproterenol therapy) (Figures 1A,B). Echocardiography excluded other abnormalities (such as coronary artery disease, congenital heart disease, and cardiomyopathy) showing normal size of ventricle and atrium, normal left ventricular systolic function with an ejection fraction (EF) of 57% and a fractional shortening (FS) of 30%. A chest radiograph showed mild pulmonary edema, enlarged heart shadow, heart-to-chest ratio of 0.57, and intestinal dilatation with gas accumulation (Figures 1C,D). Laboratory findings showed myocardial injury with elevated N-terminal brain natiruretic peptide (NT-BNP) of 13,100 pg/mL (normal range 0–100 pg/mL) and cardiac troponin I (cTnI) of 0.748 μg/L (normal range 0–0.034 μg/L). Fasting blood glucose (22.30 mmol/L, normal range 4.1–5.9 mmol/L) increased significantly. Kidney and liver enzymes also slightly increased (AST 83 U/L, ALT 60 U/L and creatinine 61 μmol/L, normal range, respectively, were 17–59 U/L,21–72 U/L,17.3–54.6 umol/L). The arterial blood gas, Complete blood count, erythrocyte sedimentation rate, rheumatism screening, and autoantibody are normal. Based on our experience, we initially considered that these conditions were chiefly caused by FM. The patient was rapidly treated with intravenous methylprednisolone (20 mg/kg per day), immunoglobulin (500 mg/kg per day), and isoproterenol (0.2 ug/kg/min). Unfortunately, bradycardia persisted after 2 days of treatment. Finally, a temporary pacemaker was inserted with a ventricular rate of 80 beats/min.

After the operation, we reviewed the past medical history of the patient and found that he was diagnosed with hypothyroidism at the age of 3 years. He was treated with levothyroxine (50 ug/d) but he had not been regularly followed up by an endocrinologist and he had withdrawn hormone supplementation before hospital admission. His mental development, motor development, and language development lagged significantly behind those of children of his age. His thyroid stimulating hormone (TSH) level increased (>150 mIU/L), and free thyroxine (FT_4_) levels decreased (1.50 pmol/L) significantly. Therefore, we wondered whether hypothyroidism could lead to a third-degree AVB and cardiogenic shock. We reviewed the relevant literature and found similar reports in adults (1–5). However, there was no such case reported in children. After careful consideration, on the second day, we administered a supplement of oral levothyroxine (50 μgd^−1^) for observation, and stopped using methylprednisolone, immunoglobulin, and isoproterenol. Surprisingly, the patient recovered well-beyond expectations. His ECG on the Holter monitor showed a sinus rhythm of 81 beats/min and the AVB disappeared on the third day from disease onset, and echocardiography revealed normal and elevated EF (66%) and FS (35%), left ventricular end diastolic diameter of 34 mm. Laboratory findings showed reduced myocardial injury markers with NT-BNP (5,870 pg/mL) and cTnI (0.135 μg/L). Clinical findings were significantly improving. As a result, we reconsidered possible causes for these conditions in the patient. Eventually, we concluded that hypothyroidism, but not FM, was the real cause of the AVB and cardiogenic shock. On the eighth day of admission, the patient was discharged on levothyroxine 50 μg daily and was advised to follow-up with cardiologist and endocrinologist for repeat thyroid function testing and ECG in 4 weeks (Figures 1E,F). During follow-up, the child's cardiac function and thyroid function returned to normal.

## Discussion

In this case report, we comprehensively discuss regarding a child with severe hypothyroidism who was misdiagnosed as having FM and whose sinus rhythm was successfully recovered after temporary pacemaker implantation and oral levothyroxine hormone supplementation. Based on our experience, we considered that the reasons for the misdiagnosis of FM were as follows: (1) The onset of the disease was sudden and the disease progressed rapidly; (2) the patient had frequent convulsions as the first symptom, followed by respiratory and cardiac arrest at another hospital; and (3) the patient developed hemodynamic instability, dull heart sounds, and bradycardia. The ECG showed a third-degree AVB, and abnormalities of other cardiac diseases were excluded by echocardiography (such as coronary artery disease, congenital heart disease, cardiomyopathy, and connective tissue diseases). However, given the ineffectiveness of intravenous methylprednisolone and immunoglobulin treatment for FM, rapid relief of the symptoms after thyroxine supplementation, slightly elevated cTnI and normal left ventricular systolic function at admission, we considered that hypothyroidism, but not FM, was the underlying cause of the AVB and cardiogenic shock. To our knowledge, this is the first case report in China of a child with a third-degree AVB and cardiogenic shock caused by hypothyroidism. This case report will improve clinician's understanding of the serious cardiovascular complications caused by hypothyroidism. In addition, we suggest that hypothyroidism should be considered as a differential diagnosis of FM.

The effects of hypothyroidism on the cardiovascular system mainly include decreased cardiac output, decreased myocardial contractility, decreased heart rate, and increased peripheral resistance (7). TH can directly promote the release of Ca^2+^ from the sarcoplasmic reticulum and activate proteins related to myocardial contraction and enhance the activity of myosin heavy chain adenosine triphosphate enzyme, thus enhancing myocardial contractility. However, TH can also increase the number and affinity of β-adrenergic receptors on the information cell membrane and improve the sensitivity of the myocardium to catecholamines. If TH synthesis is insufficient, it will lead to abnormal cardiac systolic function and cardiac electrical activity. Therefore, hypothyroidism could lead to bradycardia, dull heart sounds, reduced cardiac output, cardiac enlargement, and abnormal ECG. Although hypothyroidism could affect the cardiovascular system of patients, the symptoms and signs of cardiovascular dysfunction are not common or prominent. As a result, we did not initially consider hypothyroidism as a causative agent.

We conducted a literature review and found that there are few reported cases of cardiogenic shock and malignant arrhythmia caused by hypothyroidism, resulting in difficulties in the early identification and diagnosis. Previous reports of AVB caused by hypothyroidism have mainly been reported in adults (1–5), but rarely in children (8). Zaki (9) et al. reported a case of refractory cardiogenic shock in an infant with congenital hypothyroidism. The reasons for the rarity in children may be as follows: (1) congenital hypothyroidism can lead to developmental delay and growth disorder in children, which enables many children to receive intervention and treatment at an early stage before cardiac manifestations occur and (2) although short-term or long-term hypothyroidism does affect cardiac function, which results in cardiac dysfunction, it is not commonly seen in clinical practice and has not attracted enough attention.

Although there were few cases of cardiogenic shock and malignant arrhythmias caused by hypothyroidism seen in our literature review, we found that timely thyroxine supplementation in the early stage of the disease can significantly improve the prognosis of adults in the short term. In view of our experience and this information, we reported this case in order to raise the awareness of our colleagues regarding this rare phenomenon. When we diagnose FM in children, in addition to excluding other diseases, we should rule out metabolic causes such as hypothyroidism, thereby avoiding delays in the diagnosis and leading to prompt treatment.

## Conclusions

Hypothyroidism is a common disease in clinical practice, but secondary cardiovascular lesions are rare. The delay in diagnosis is quite serious because of the low awareness and recognition of the situation. This will continue to be challenging in the future, especially in developing countries. Based on comprehensive history collection, when patients develop severe AVB and bradycardia, we need to consider hypothyroidism as a possible causative factor. To our knowledge, our case is the first report in the Chinese population of hypothyroidism in a child misdiagnosed with FM. We hope that our findings benefit other pediatricians when managing patients with cardiogenic shock and AVB.

## Data Availability Statement

The original contributions presented in the study are included in the article/supplementary material, further inquiries can be directed to the corresponding authors.

## Ethics Statement

Written informed consent was obtained from the individual(s), and minor(s)' legal guardian/next of kin, for the publication of any potentially identifiable images or data included in this article.

## Author Contributions

NZ and SS drafted the manuscript, contributed to the case collection, provided Figures 1A–D, and approved the final manuscript as submitted. YY contributed to the study design and approved the final manuscript as submitted. YH provided Figures 1E,F, funding support, and approved the final manuscript as submitted. KZ and CW provided major treatment on the patient while admitted, provided financial support, and approved the final manuscript as submitted. All authors contributed to the article and approved the submitted version.

## Conflict of Interest

The authors declare that the research was conducted in the absence of any commercial or financial relationships that could be construed as a potential conflict of interest.

## Abbreviations

AVB: atrioventricular block
cTnI: cardiac troponin I
ECG: electrocardiogram
EF, ejection fraction;FM: fulminant myocarditis
FS: fractional shortening
FT_4_: free thyroxine
Mb: myohemoglobin
NT-BNP: N-terminal brain natiruretic peptide
TSH: thyroid stimulating hormone.
